# Imaging pathology in archived cornea with Fuchs’ endothelial corneal dystrophy including tissue reprocessing for volume electron microscopy

**DOI:** 10.1038/s41598-024-82888-5

**Published:** 2024-12-30

**Authors:** Sayo Maeno, Philip N. Lewis, Robert D. Young, Yoshinori Oie, Kohji Nishida, Andrew J. Quantock

**Affiliations:** 1 Department of Ophthalmology, Osaka University Graduate School of Medicine, Suita, Osaka Japan; 2 Structural Biophysics Research Group, School of Optometry & Vision Sciences, Cardiff University, Cardiff, Wales, UK

**Keywords:** Cornea, Fuchs’ endothelial corneal dystrophy, Archive tissue reprocessing, Volume electron microscopy, Structural biology, Diseases

## Abstract

Fuchs’ endothelial corneal dystrophy (FECD) is a common sight-threatening condition characterised by pathological changes in the posterior cornea. Here we report observations by light, transmission and volume scanning electron microscopy on changes in the endothelium and matrix associated with the characteristic deformations of Descemet’s membrane, termed guttae. Specimens were archived full-thickness human corneal tissue, removed during graft surgery, that had been fixed, stained and embedded by conventional processing methods for examination by transmission electron microscopy more than 40-years previously. Intact archived samples can be extremely valuable where, as with FECD, new cell-based methods of therapy now avoid excision of the full cornea thickness and any tissue excised is inferior for study. Volume electron microscopy, in particular serial block face scanning electron microscopy (SBF SEM), employing backscatter electron detection from resin-embedded specimens, has become an invaluable technique for 3D imaging of biological samples. However, archived specimens are normally considered unsuitable for imaging as conventional processing methods generate low backscatter electron yield. To overcome this for SBF SEM, we subjected epoxy resin-embedded specimens to de-plastination, then applied additional contrasting agents, uranyl acetate and lead acetate, prior to re-embedding. Selected regions of interest in the new resin blocks were examined in a scanning electron microscope equipped for SBF SEM and serial image datasets acquired. Enhanced contrast enabled 3D reconstruction of endothelium and guttae in Descemet’s membrane over large tissue volumes.

**Supplementary Information:**

The online version contains supplementary material available at 10.1038/s41598-024-82888-5.

## Introduction

Fuchs’ endothelial corneal dystrophy (FECD) is a bilateral, sight-threatening disorder characterized by the abnormal production of extracellular matrix by the corneal endothelium, typically causing formation of excrescences, termed guttae, in the adjacent Descemet’s membrane and progressive corneal oedema. The resulting, irreversible corneal endothelial dysfunction leads to reduced corneal transparency and subsequent visual impairment^1–5^. FECD is the most common reason for corneal transplantation worldwide^6^, accounting for 39% of keratoplasties in a survey of 116 countries^7^, yet its aetiology is still not fully understood. Currently, there is considerable interest in the mechanisms underlying FECD, with recognition of early and late-onset variants and intense focus on the heterogeneous genetic basis underpinning some forms, alongside an acknowledgement that cases without defined inheritance are most common^8,9^. Evidence is accumulating for the involvement of diverse, interacting pathogenic mechanisms in the development of FECD, including oxidative stress^10,11^, cell death via apoptosis^12,13^, endoplasmic stress and the unfolded protein response^14,15^ and epithelial-mesenchymal transition^16,17^. Current weight of opinion, however, appears to favour a disorder of mitochondrial function to be a key factor^9,18–20^.

Traditionally, surgical treatment of FECD was via penetrating keratoplasty, replacing a full-thickness of the central cornea with donor tissue, but this has now been largely superseded by endothelial keratoplasty, performed on 94.5% of FECD patients undergoing surgery in the USA in 2020^21^. Various forms of endothelial keratoplasty and also descemetorhexis without endothelial keratoplasty are in use for FECD^22^. Some involve transplantation of donor stroma, Descemet’s and endothelium directly onto the recipient posterior corneal surface, others applying donor Descemet’s and endothelium, or cultured cells on a carrier substrate, to the recipient, after removal of the host membrane and cells^23–27^. Thus, intact excised material for structural studies of morphological events in FECD progression is becoming increasingly scarce. Consequently, archived samples derived from traditional, full-thickness surgeries are extremely valuable.

Specular microscopy and laser scanning confocal microscopy are effective tools for in vivo imaging of the posterior corneal surface in FECD patients^28,29^. However, higher resolution analyses require electron optical methods applied to excised samples^30,31^. Recently, volume electron microscopy, which uses backscatter electron detection from highly contrasted resin-embedded specimens, has become an invaluable technique for 3D imaging of biological samples^32^. However, archived specimens are normally considered unsuitable for imaging as the lesser amounts of high contrast reagents used in conventional processing methods generate low backscatter electron yield. We report observations by light and transmission electron microscopy and also by serial block face scanning electron microscopy (SBF SEM) on the posterior cornea in archived FECD specimens obtained in the 1980s. For SBF SEM, specimens were first exposed to a solution of sodium ethoxide to remove the original resin. Additional contrasting reagents were then applied and the tissue re-embedded. A similar approach has been reported previously to remove resin from epoxy sections for histological staining for light microscopy^33^; from embedded tissue before critical-point-drying for conventional scanning electron microscopy^34^; and to etch ultrathin resin sections to expose epitopes for immunogold labelling^35^. We then acquired serial image datasets, permitting reconstructions to be made of posterior cornea and guttae in 3D. With further refinement this procedure should prove widely applicable for the examination of archived biological specimens of diverse specification for SBF SEM.

## Results

### Light microscopy

**Fig. 1:**
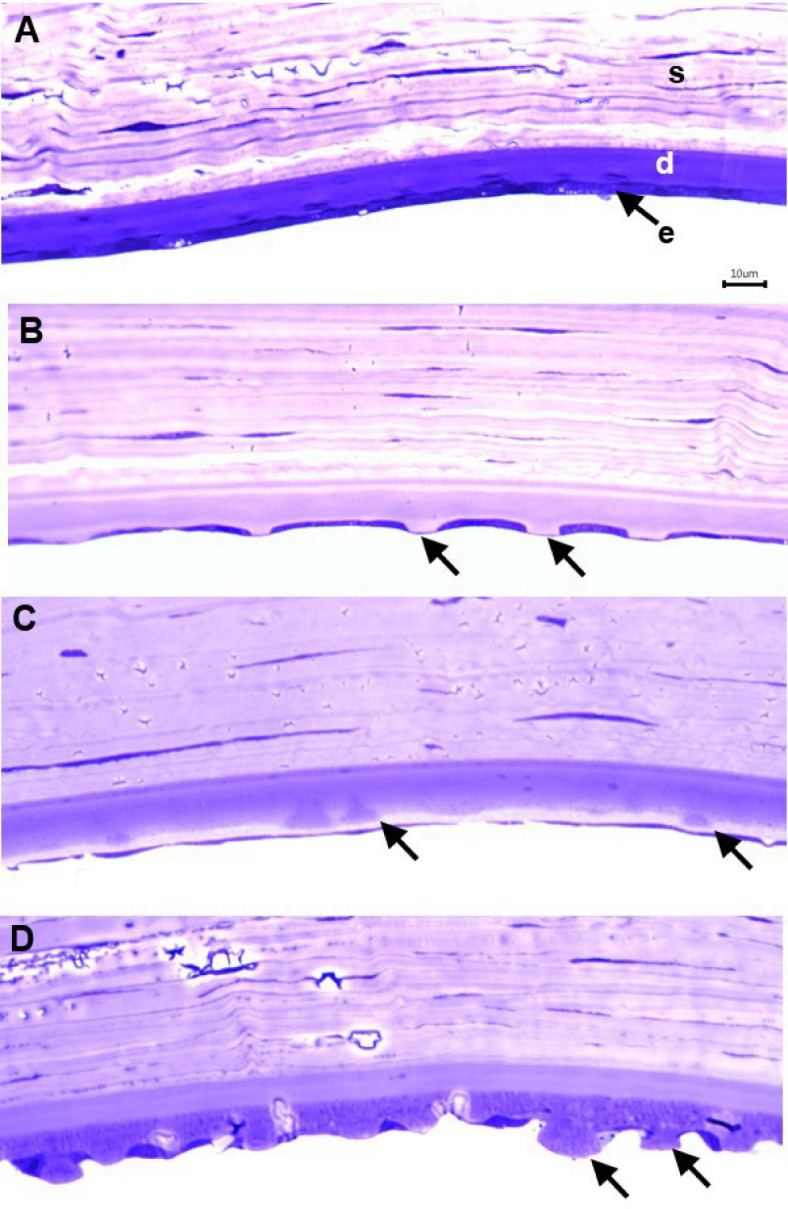
Toluidine blue-stained semi-thin sections of posterior cornea. (**A**) Normal cornea showing regular thickness of Descemet’s membrane and endothelial cell layer. (**B**–**D**) Corneas from patients with FECD. (**B**) Attenuation of endothelial cells overlying guttae in Descemet’s membrane (arrows) in an 81-year-old female patient. (**C**) In a 64-year-old female FECD patient, guttae appear embedded within new matrix (arrows), presumably synthesised by intact endothelial monolayer. (**D**) Advanced guttae formation (arrows) in an 81-year-old male with FECD, together with interruption of endothelial monolayer and thickening of Descemet’s membrane. s, stroma; d, Descemet’s membrane; e, endothelium.

Preliminary examination of specimens was carried out before reprocessing for volume electron microscopy. Initial light microscopy on toluidine blue-stained, semi-thin sections enabled identification of locations in posterior cornea suitable for subsequent high-resolution imaging (Fig. 1). The specimen from a normal donor eye exhibited regular thickness of Descemet’s membrane and proximal endothelial monolayer (Fig. 1A). Three specimens from patients with FECD showed varying extents of guttae formation in Descemet’s membrane, including protuberances surfaced by attenuated endothelium (Fig. 1B), guttae embedded within an apparent repair extracellular matrix with intact endothelial monolayer (Fig. 1C) and extensive guttae formation on much-thickened, Descemet’s membrane with discontinuous endothelial cells (Fig. 1D).

### Transmission electron microscopy

**Fig. 2:**
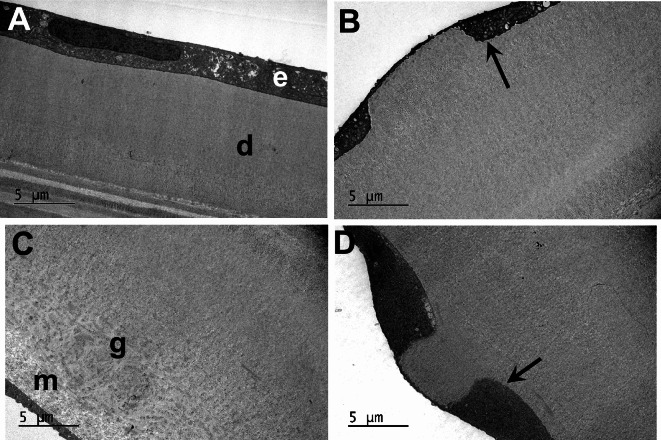
TEM across endothelium and Descemet’s membrane to posterior stroma. (**A**) endothelium (e) and Descemet’s (d) exhibit regular thickness in normal cornea; (**B**–**D**) in FECD, thickened Descemet’s with attenuated endothelium is evident, although cell layer deepens (arrows) around prominent guttae, which project proximally. (**C**) A proximal fibrous layer of matrix (m) is present around a gutta (g) in Descemet’s membrane.

**Fig. 3:**
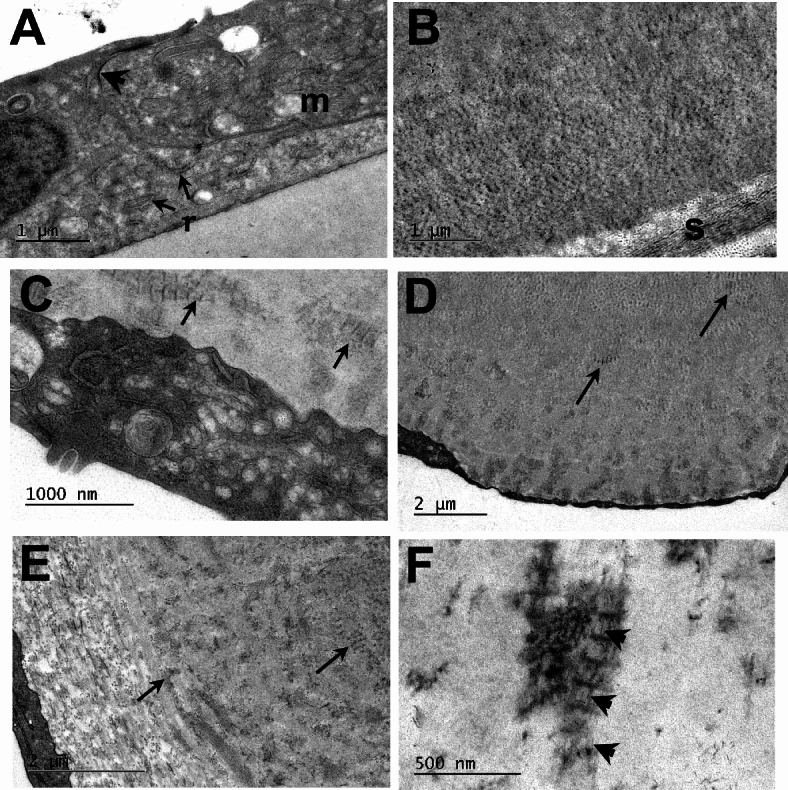
TEM reveals ultrastructural details of normal and FECD endothelium and Descemet’s membrane. (**A**,** B**) Intercellular tight junctions (arrowhead), RER (r) and mitochondria (m) are characteristic of normal corneal endothelium; a banded matrix component is conspicuous in the distal layer of Descemet’s membrane subjacent to posterior stroma (s). (**C**–**F**) in FECD where endothelium is non-attenuated, cells reveal degeneration of mitochondria, scarce tight junctions and cytoplasmic vacuoles. Long-spaced, banded matrix is common adjacent to cells (arrows), and evident both in classic guttae (**D**) and where a posterior fibrous layer is present (**E**). (**F**) Cuprolinic blue-positive filaments (arrowheads) are associated with long spaced fibrous structures.

The regular thickness of endothelial cell monolayer and Descemet’s membrane with distal banded and proximal non-banded components were evident in normal cornea at higher resolution, but were replaced in FECD by an attenuated cell layer and thickened membrane with prominent sculpted guttae extending proximally (Fig. 2). Normal endothelial cells are characterised by the presence of intercellular tight junctions together with numerous mitochondria, profiles of rough endoplasmic reticulum (RER) and Golgi, typical of dynamically active cells (Fig. 3A). A layer of banded matrix is also conspicuous in the anterior region of Descemet’s membrane (Fig. 3B). Where intact endothelium was located in FECD intercellular tight junctions were rarely identified and the cytoplasm was dominated by degenerate mitochondria and vacuoles (Fig. 3C); in addition, banded matrix structures were present adjacent to the cells and commonly observed throughout guttae (Fig. 3D,E). Fibrillar and banded matrix structures had a heterogeneous appearance especially in a specimen where a posterior fibrillar layer was deposited around guttae (Fig. 3E). Banded structures resembled long spacing collagen and were associated with cuprolinic blue-positive filaments, presumed to be proteoglycans (Fig. 3F).

#### SBF SEM

**Fig. 4:**
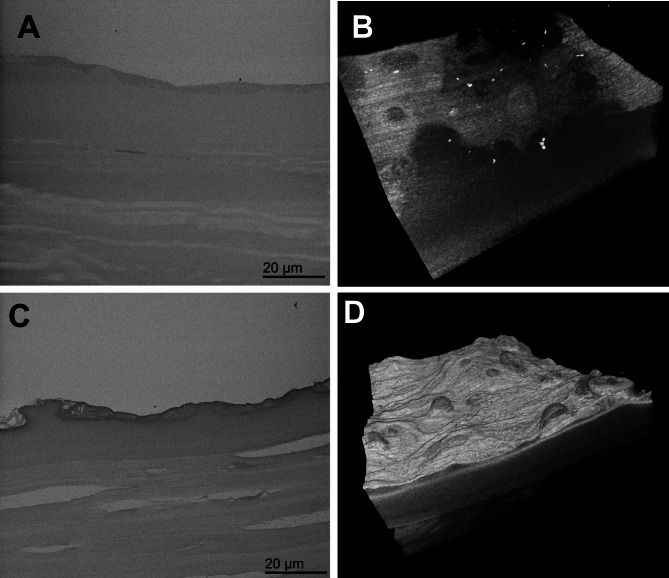
Comparison under identical imaging conditions between SBF SEM on an archived FECD specimen without or with application of the re-processing protocol. Raw data image (**A**) and 3D reconstruction (**B**) without reprocessing; Raw data image (**C**) and 3D reconstruction (**D**) after reprocessing. The block imaged derived from the same FECD specimen as that illustrated in Fig. 6.

**Fig. 5:**
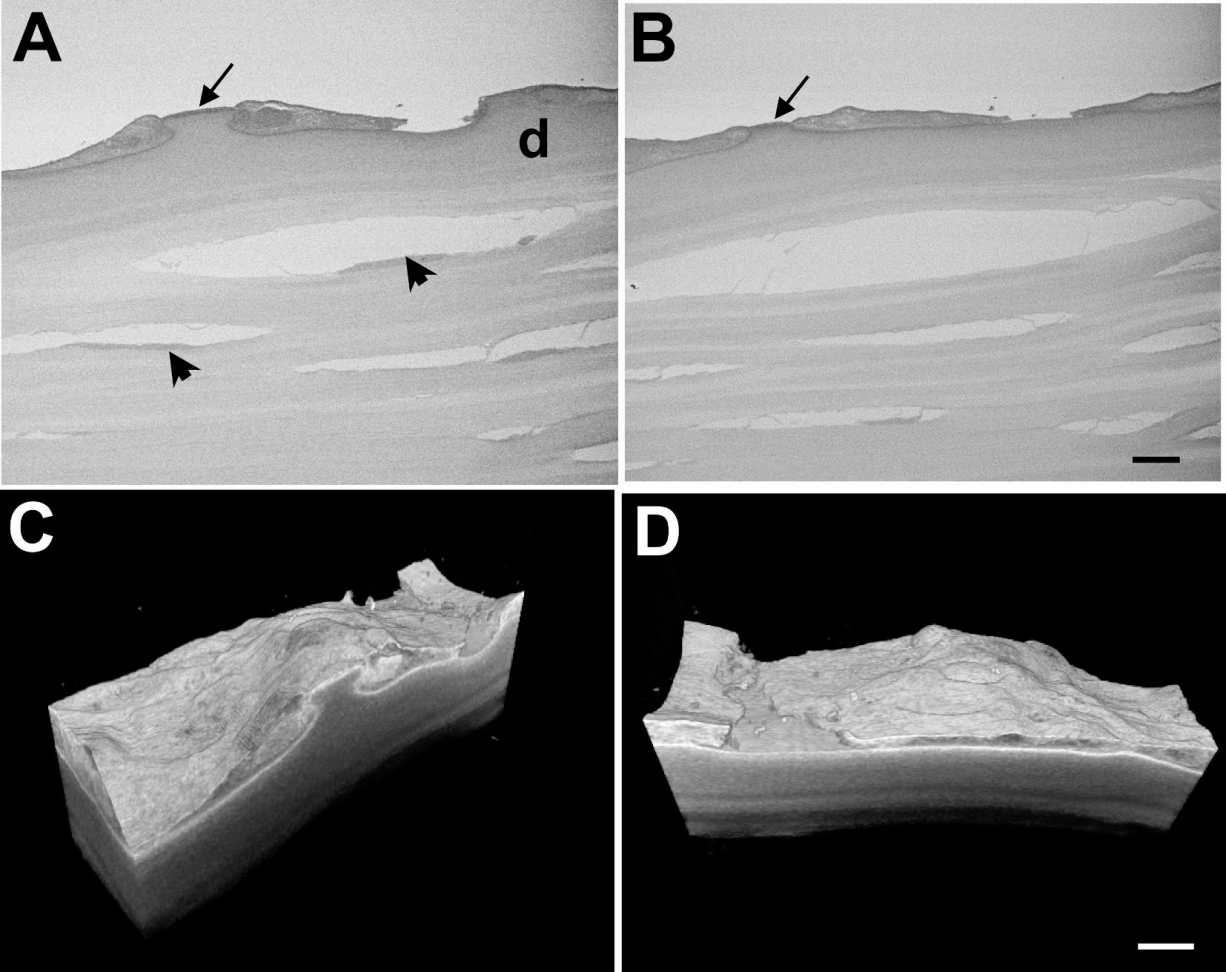
Images showing raw data and 3D reconstructions from SBF SEM after re-processing an archived specimen from an 81-year-old female FECD patient. Raw data, images 014 (**A**) and 387 (**B**), from a 505 serial image dataset,showing endothelium is attenuated at sites where guttae are forming(arrows), but is elsewhere intact over swollen Descemet’s membrane (d).Keratocytes are visible alongside artefactual splits in stromallamellae (arrowheads). (**C**) and (**D**) show stills taken from Supplementary video S1 of a 3D reconstruction of the dataset made in Amira. Bar, 10μm.

**Fig. 6:**
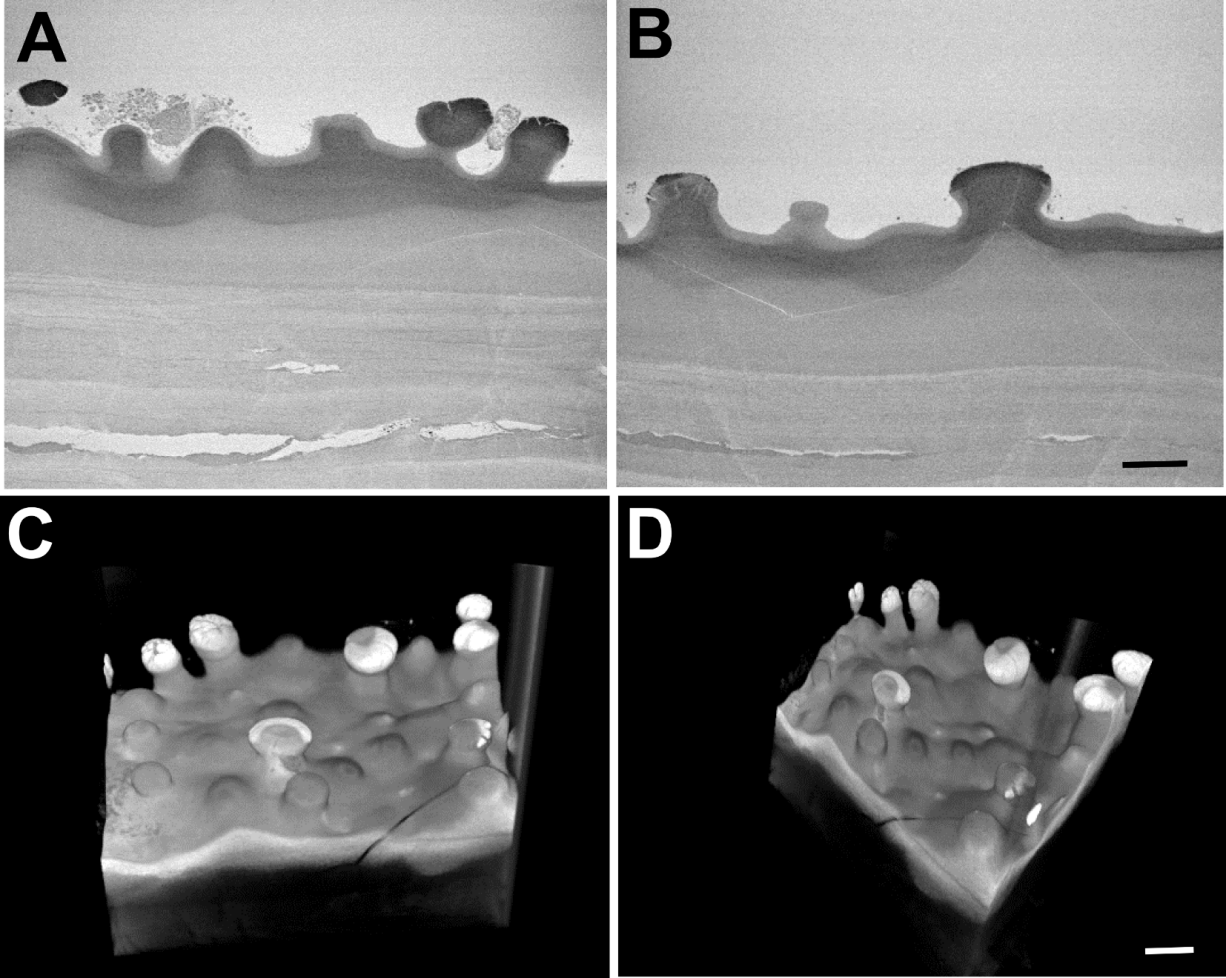
Images showing raw data and 3D reconstructions from SBF SEM after re-processing an archived, advanced FECD specimen from an 81-year-old male patient. Raw data, images 049 (**A**) and 232 (**B**), from a 571 serial image dataset show prominent and extensive guttae on Descemet’s membrane with loss of endothelial cells. (**C**) and (**D**) show stills taken from Supplementary video S2 of a 3D reconstruction of the dataset indicating extensive remodelling of thickened Descemet’s membrane. Bar, 10 μm.

Initial attempts to image surface structure in archive blocks by backscattered electron detection proved unsuccessful with little detail discernible. Observation under identical imaging conditions after application of a sample reprocessing technique demonstrated considerably improved contrast (Fig. 4). Re-processing of specimens provided sufficient contrast to enable imaging by SBF SEM. Raw serial images (Figs. 5A,B and 6A,B) and 3D reconstructions (Figs. 5C,D and 6C,D), made using automated volume rendering with Amira software, illustrated variations in the extent of guttae formation in different specimens. These ranged from thickening of Descemet’s membrane with largely intact endothelial layer and relatively few guttae in a cornea from an 81-year-old female patient (Fig. 5; Supplementary Video 1), to considerable deformation of Descemet’s membrane, substantial endothelial cell loss and presence of multiple guttae in a specimen from an 81-year-old male with reported corneal decompensation (Fig. 6; Supplementary Video 2).

## Discussion

Archived resin-embedded specimens of human cornea obtained from penetrating keratoplasty on three patients with FECD were examined by light and transmission electron microscopy then used to investigate a technique of reprocessing to render them suitable for imaging by SBF SEM. This successfully enabled acquisition of serial images with sufficient contrast for generation of 3D reconstructions demonstrating the significant restructuring of Descemet’s membrane in advanced-stage disease. All specimens were originally collected nearly 40 years ago predating recent classifications of the condition based on new gene-linkage analysis and disease severity^8,36^. Most FECD-associated genes so far identified appear linked to late-onset disease^26^, for example, *TCF4* coding for the helix-loop-helix transcription factor E2-2^37–40^, *LOXHD1*^41^ and *SLC4A11*^42^, both coding for plasma membrane proteins and the transcription factor coding gene, *TCF8/ZEB1*^16,43,44^. Early-onset FECD has been linked to *COL8A2* which codes for the α2 chain of collagen type VIII, which is a known component of Descemet’s membrane^45,46^. Mutations in these genes may all give rise to morphological changes through direct or indirect effects on aspects of connective tissue matrix production.

Findings from TEM revealed the presence of long-spacing banded matrix within Descemet’s membrane with degeneration of mitochondria, accumulation of cytoplasmic vacuoles and scarce tight junctions in the endothelial cells, consistent with previous studies^18,20,30,47^. Thickened Descemet’s membrane, containing long-spacing collagen with associated cuprolinic blue-positive filaments was present in all specimens. However, overall, three quite distinct pathological presentations were evidenced in the specimens studied here: these ranged from extreme deformation of Descemet’s membrane with the presence of large sculpted guttae and extensive loss of endothelium; through relatively few guttae and endothelium intact, but attenuated; to intact endothelium and guttae embedded in a posterior fibrous layer. Thickening of Descemet’s membrane was present in all specimens. It cannot be known whether these different presentations represent different underlying genetic basis or are merely different stages in a common disease progression. Nevertheless, for the purpose of this preliminary study it is encouraging that the reprocessing method applied successfully enabled SBF SEM imaging to be undertaken in all cases.

Our underlying premise was that insufficient backscatter electron yield for SBF SEM, which arises from the lack of contrasting agents used in traditional methods of tissue processing, could be remedied by reprocessing archive specimens. For this, preliminary removal of embedding resin surrounding the tissue is essential. Ethanolic sodium ethoxide was the reagent selected for this purpose on account of its use previously to de-plastinate resin sections to expose protein epitopes for immunogold labelling for transmission electron microscopy. An earlier comprehensive study of the effects of the reagent at different concentrations, measured by the efficacy of immunogold labelling^35^ on treated ultrathin sections, provided a useful guide to the most suitable concentration for resin removal from whole tissue blocks. Ethoxide reagent is an aggressive solvent with potential to remove elements of the tissue with prolonged exposure. Reduction in gold label density at some levels of ethoxide exposure however was previously interpreted as an indication of degradation of sample structure^35^. This might render attempts to expose structures situated distant from the surface less likely to succeed without loss of surface material. Small, localised amounts of residual resin, observed in samples treated in this study, we believe indicated that excessive surface tissue damage was avoided. We are optimistic that the approach has application for use in a wide range of archived samples, but preliminary optimisation for individual specimens is recommended. Further development of the technique is required perhaps with initial exposure of structures of interest at deeper subsurface locations by microtomy prior to ethoxide application. Inclusion of additional re-contrasting solutions, such as osmium tetroxide, may also be worthwhile to enhance imaging above that achieved in the study presented here.

The instrument used for imaging in this study was a scanning microscope operating under variable pressure conditions in which the whole chamber receives an operator-selected level of dry nitrogen gas. The nitrogen is fundamental to detection of the backscatter electron signal and reduces build-up of electrical charge at the specimen surface, which can affect image resolution. More recent developments of the technology provide a system for focal charge compensation, where limited controlled nitrogen gas is passed directly onto the specimen surface, negating the need for whole chamber gas inclusion. This in turn allows high vacuum and greater beam energy operation, which contribute to more efficient signal detection and less charging artefacts. It is quite likely that some archived specimens may be effectively imaged by this method without the need for reprocessing and contrast enhancement. However, sample re-processing is available as an option where imaging still fails to generate adequate contrast.

In conclusion, this study confirms that de-plastination, contrast enhancement and resin-re-embedding of conventionally processed TEM specimens enables high quality image data to be collected by volume SEM. Reconstructions made from the datasets from FECD corneas examined here provide a revealing three-dimensional perspective on the extent of Descemet’s membrane remodelling in this condition. This approach offers considerable promise for future studies of rare archived clinical specimens.

## Methods

### Human cornea samples

This study used existing holdings of ocular tissue samples, obtained prior to the commencement of the Human Tissue Act (HTA, 2006), and with full NHS Research Ethics Committee Approval (REC reference: 21/NS/0091). The full-thickness corneal tissue used in the study was originally obtained in the mid 1980’s from two female patients (aged 64 and 81 years) and one male with associated corneal decompensation (aged 81 years), undergoing penetrating keratoplasty in treatment of FECD. Normal corneal tissue for comparison was obtained from a donor eye unsuitable for use in transplantation. Informed consent was obtained from all patients prior to surgery and the research was performed in accordance with the tenets of the Declaration of Helsinki and under HTA licence-approved standard operating procedures in Cardiff University’s School of Optometry and Vision Sciences.

Specimens were processed by conventional methods for transmission electron microscopy, including fixation in 2.5% glutaraldehyde in 0.1 M sodium cacodylate buffer for 5 h, postfixation in 1% aqueous osmium tetroxide, then 1% aqueous uranyl acetate, each for 1 h, before dehydration in ethanol and embedding in Araldite CY212 epoxy resin. The blocks thus produced were stored at room temperature in the dark until further preparation described below. Some tissue from one patient was fixed in aldehyde solution in the presence of the cationic dye, cuprolinic blue to permit visualisation of proteoglycans, before embedding in epoxy resin.

### Light and transmission electron microscopy

Semi-thin sections, approximately 0.3 μm thick, of central cornea were first cut on an ultramicrotome and stained with 1% toluidine blue in 1% sodium tetraborate for identification of regions of interest by light microscopy. Blocks were trimmed to a cutting face of around 0.4 × 0.2 mm and ultrathin sections, 90 nm thick collected on copper grids for contrasting with saturated aqueous uranyl acetate and lead citrate solutions, before examination in a transmission electron microscope.

### Reprocessing of specimens for SBF SEM

Excess resin was removed from around the specimen with a razor blade taking care to avoid damage to the posterior face of the cornea. Specimens were then immersed in 4% or 7% sodium ethoxide in ethanol (diluted from 21% commercial reagent; Cat. No.10568505, Fisher Scientific, Loughborough, UK) in sealed glass vials on a rotator for 6–24 h. Stock sodium ethoxide is highly flammable and causes burns on skin contact and thus must be handled wearing protective clothing in a fume hood, away from sources of heat and ignition. When observation with a binocular dissecting microscope confirmed that the resin had been removed, the specimens were rinsed in several changes of ethanol over 48 h before immersion in 1% ethanolic uranyl acetate for 16 h. Further washes in ethanol, then overnight immersion in 1:1 ethanol: acetone mixture, were followed by 4 h exposure to lead acetate in ethanol: acetone and final rinsing in 100% acetone. The specimens were then re-embedded in Araldite CY212 resin. Briefly, this involved an extended period of resin re-infiltration, first with resin mixture lacking Accelerator, before application of the full resin mixture and polymerisation at 60 °C over 4 days in total, according to an established procedure for SBF SEM^48^.

### SBF SEM

Blocks were trimmed and glued to aluminium specimen carriers to provide a cutting face perpendicular to the plane of the corneal endothelium and regions of interest located as described above. Conductive carbon cement was applied to exposed resin surfaces around the sides of the block and allowed to air-dry thoroughly before application of a 14 nm layer of gold in a vacuum sputter coater (EM ACE 200, Leica Microsystems). SBF SEM imaging, data acquisition and post-data processing were followed, as previously described^49^. A scanning electron microscope (Sigma VP FEG, Carl Zeiss, Cambridge, UK) with integral serial block face capability (3View2, Thermo Fisher Scientific-Gatan Inc, Pleasanton, USA) operating at 3.5 kV, 25 Pa dry nitrogen gas pressure and 8 msec beam dwell time was used to capture 400–570 image sequences at 100 nm intervals into the block, at 800x magnification and a scan resolution of 4096 × 4096 pixels, with 45 nm/pixel. 3D reconstructions were made from the serial image datasets by automated volume rendering using Amira 2022.2 software (Thermo Fisher Scientific, Hemel Hempstead, UK) to illustrate the morphology of the posterior corneal stroma, Descemet’s membrane and endothelium.

## Electronic supplementary material

Below is the link to the electronic supplementary material.

Supplementary Material 1

Supplementary Material 2

Supplementary Material 3

Supplementary Material 4

## Data Availability

The SBF SEM datasets generated during the current study are available from the corresponding author on reasonable request.
