# Supplementary material for: Imaging pathology in archived cornea with Fuchs’ endothelial corneal dystrophy including tissue reprocessing for volume electron microscopy
Source: Sci Rep. 2024 Dec 30;14:31786. doi: 10.1038/s41598-024-82888-5 (PMC11685999; doi:10.1038/s41598-024-82888-5)
Supplement: Supplementary file 1 — Supplementary Material 1 [file 41598_2024_82888_MOESM1_ESM.pdf]

Supplementary Information: Methodology

Manuscript Submission ID fd194d9c-14bd-4dbe-9d79-d6de932fcccf
(Revised title)

Imaging pathology in archived cornea with Fuchs endothelial corneal dystrophy including tissue reprocessing for volume electron microscopy

Sayo Maeno, Philip N. Lewis, Robert D. Young, Yoshinori Oie, Kohji Nishida and Andrew J. Quantock

Reprocessing of archived epoxy resin-embedded tissue for SBF SEM

1. General Comments

The procedure should be conducted in a laboratory with equipment and expertise in sample processing for electron microscopy,. This setting ensures the implementation of risk assessments and adherence to standard operating procedures, ensuring the safe handling and proper disposal of all reagents.

Specimens prepared by conventional methods for TEM, are treated to remove resin and techniques originally established for SBF SEM including BSE contrasting, epoxy resin re-embedding and re-polymerization, for ultramicrotomy applied. A fume hood is crucial for conducting staining and resin embedding steps, as the chemicals involved pose toxicity risks through inhalation and skin contact. When handling hazardous chemicals and solvents, it is recommended to use solvent-resistant NBR 92-600 gloves, and wearing laboratory coats provides additional protection against chemical exposure.

The protocol requires 9 days to remove the original resin, stain, infiltrate with resin and embed specimens in moulds.

1.1 Materials

- Sodium ethoxide, 21% in ethanol, AcroSeal (Thermo Scientific, Stansted, UK; Catalogue No: 429301000)
- Uranyl Acetate (TAAB Laboratories, Aldermaston, UK; Catalogue No. U007)
- Lead Acetate (Agar Scientific, Stansted, UK; Catalogue No: AGR1209)
- Absolute ethanol 99%+, extra pure SLR (Fisher Chemical E/0600DF/17)
- Acetone 99.5%, laboratory reagent (Fisher Scientific UK Ltd., Loughborough, UK; Catalogue No: 179973-1L)
- Araldite CY212 epoxy resin (Agar Scientific, Stansted, UK; Catalogue No: AGR1040)
- DDSA (Dodecenyl Succinic anhydride) resin hardener (Agar Scientific, Stansted, UK; Catalogue No: AGR1051)
- BDMA (Benzyl dimethylamine) Accelerator (Agar Scientific, Stansted, UK; Catalogue No: AGR1062)
- 7 mL glass specimen vials (Agar Scientific, Stansted, UK; Catalogue No: AGG284)
- Flat silicone embedding mould (Agar Scientific, Stansted, UK; Catalogue No: AGG3549)
- Luer-lock (optional) syringes 10 mL BD PlastiPak (Fisher Scientific UK Ltd., Loughborough, UK; Catalogue No: 15544835)
- 0.2 um Syringe filters, Sartorius Minisart™ RC (Fisher Scientific UK Ltd., Loughborough, UK; Catalogue No: 11740966)
- Plastic transfer pipettes, Fisherbrand™1 mL (Fisher Scientific UK Ltd., Loughborough, UK; Catalogue No: 13439118)
- Plastic transfer pipettes, Fisherbrand™3 mL (Fisher Scientific UK Ltd., Loughborough, UK; Catalogue No: 16405009)

1.2 Equipment

- Hot Plate Stirrer, Fisherbrand™ (Fisher Scientific UK Ltd., Loughborough, UK; Catalogue No: 15363518)
- Low speed Rotator (Agar Scientific, Stansted, UK; Catalogue No: AG1050)
- Vortex mixer (Starlab, Milton Keynes, UK; N2400-6110)
- Embedding oven, placed inside fume hood (Agar Scientific, Stansted, UK; Catalogue No: AGB7606)

2. Procedure

Place the samples in 4% or 7% Sodium ethoxide in 100% ethanol for 6-24 hrs. Wash the samples by replacing the buffer with 3 mL of fresh 100% ethanol to remove any traces of residual reagents. Then, replace with another 100% ethanol for 3-4 days.

PAUSE STEP The samples can be stored in 100% ethanol in a refrigerator at 4℃.

2.1 *En bloc* staining

CRITICAL STEP Before resuming the protocol, make up 1% ethanolic uranyl acetate solution in a fume hood before use. Prepare sufficient amounts of each for 3 mL per sample vial. 1% ethanolic uranyl acetate solution is prepared by directly weighing powder into a 50 mL solvent-resistant polypropylene universal tube and adding ethanol. Seal the cap securely with parafilm to prevent any potential leakage. Vortex the solution until the powder is dissolved, followed by wrapping the tube in aluminium foil for storage at room temperature in the dark. Prior to use, draw the solution up into a 20 mL Luer-lock syringe and attach a sartorius RC 0.20 um syringe filter.

Remove 100% ethanol from the samples and add the filtered 1% solution of uranyl acetate in ethanol overnight.

CRITICAL STEP The addition of 1% alcoholic uranyl acetate overnight further enhances backscatter electron contrast improving overall imaging quality for SBF SEM data acquisition.

On the following day, rinse the samples in ethanol for two times 3hrs each to remove excess uranyl acetate. Next, remove ethanol and place in 1:1 100% ethanol and acetone solution overnight.

CRITICAL STEP Before starting the following day, prepare fresh lead acetate solution. Prepare the lead acetate solution by adding 1.4g of lead acetate to 25 mL of 100% ethanol in a 50 mL plastic universal tube. Seal the tube tightly with a screw-top plastic cap, and further secure it by wrapping parafilm around the cap to prevent leakage. Vortex the solution and gently shake the tube intermittently for 15 min. Afterwards, introduce 25 mL of 100% acetone to the ethanolic solution, reseal the cap with parafilm, and continue vortex and shaking for an additional 15 min. Filter the mixture using Whatman 1 filter paper to eliminate solid particles, collecting the clear solution in a clean 50 mL universal tube. Finally, draw up the lead solution into a 20 mL Luer-lock syringe and attach a 0.2 µm syringe filter.

Remove 1:1 100% ethanol and acetone solution, and add the lead acetate solution for 4hrs. After lead *en bloc* staining, wash in 1:1 ethanol acetone solution for 1hr. The 1:1 ethanol acetone solution is then replaced with 100% acetone for 2hrs, followed by the beginning of resin infiltration overnight.

CRITICAL STEP The transitional step involving 100% acetone is crucial for eliminating any trace of ethanol, as ethanol is not compatible with CY212 Araldite epoxy resin. The presence of any residual ethanol could impede successful polymerization of resin blocks for SBF SEM.

2.2 Resin Infiltration

CRITICAL STEP Resin infiltration is performed for the same duration as that for volume electron microscopy sample processing, as the heavy metal solutions used in the previous steps make

the sample extremely dense. To prolong the infiltration process, the sample undergoes a two-stage exposure to the embedding resin: initially without the BDMA accelerator, followed by the second stage with the complete resin mixture, including the accelerator.
Prepare a sufficient amount of Araldite resin mixture for the entire embedding procedure, as a second batch may not uniformly integrate with one made earlier in which crosslinking will be already advanced. The full resin mixture contains the 3 components in the following proportions:

Araldite CY212 monomer	14 mL
DDSA Hardener	16 mL
BDMA Accelerator	0.6 mL

Determine the volume required, estimating approximately 3 mL per sample vial per change; as a general guideline, allow for a total of 12 resin changes, 6x without and 6x with BDMA accelerator. This number is not absolute, adjusting for smaller specimens or increasing for sizeable or dense tissue samples. A small excess should also be added to accommodate the intermediate acetone: resin step (described below), and also for sufficient medium to fill embedding mould wells.

Preheat the monomer and hardener by placing CY212 abd DDSA reagent bottles in the embedding oven for 30 min at 60℃, to facilitate the measuring and pouring process. Additionally, prewarm a measuring cylinder and receiving glass conical flask to enhance efficient dispensing and mixing of the two components. Combine both in a suitable measuring cylinder, and then transfer to the warmed flask, manually swirling for several minutes to ensure thorough mixing of the contents.

Make up sufficient 1:1 mixture of 100% acetone and resin without BDMA to allow about 3 mL per specimen vial and transfer to vials for overnight, on the rotator with vial caps in place.

On the following day, make five changes of the resin without accelerator at intervals of approximately 1 h, replacing vial caps after every exchange. Switch then to carry out four 2h changes with the full resin with BDMA mixture, replacing vial caps after every exchange. With the last resin change of the day, remove caps from the vials and leave on rotation overnight.

On the following day, perform an additional three further 2h changes of full resin mixture before transferring the samples to blue silicon rubber EM moulds.

CRITICAL STEP For embedding the specimens, place the flask with residual resin in the embedding oven for 10–15 min before introducing the specimens into the wells of the mould. This reduces resin viscosity, and will assist precision in applying small amounts by pipette into the mould wells. Orient the samples in the mould for an appropriate sectioning plane for 3view imaging, adjusting the specimens with fine forceps under a dissecting microscope in the fume hood. Immerse labels with specimen details in the resin, keeping them clear of the sample and prospective cutting face. Top up individual wells with sufficient extra resin for a level surface. The mould with specimens is then put into an embedding oven in the fume hood. Polymerize the resin at 60 °C for a minimum of 24 h.
