# Supplementary material for: Imaging pathology in archived cornea with Fuchs’ endothelial corneal dystrophy including tissue reprocessing for volume electron microscopy
Source: Sci Rep. 2024 Dec 30;14:31786. doi: 10.1038/s41598-024-82888-5 (PMC11685999; doi:10.1038/s41598-024-82888-5)
Supplement: Supplementary file 4 — Supplementary Material 4 [file 41598_2024_82888_MOESM4_ESM.docx]

**Legends for Supplementary Videos**

**Supplementary Video S1**

Video S1. Related to Figure 5. 3D reconstruction from SBF SEM after re-processing an archived specimen from an 81-year-old female FECD patient with forming guttae over swollen Descemet’s membrane.

**Supplementary Video S2**

Video S2. Related to Figure 6. 3D reconstruction from SBF SEM after re-processing an archived, advanced FECD specimen from an 81-year-old male patient, displaying prominent and widespread guttae on Descemet's membrane accompanied by a loss of endothelial cells.
